# Inspiratory Muscle Training Program Using the PowerBreath^®^: Does It Have Ergogenic Potential for Respiratory and/or Athletic Performance? A Systematic Review with Meta-Analysis

**DOI:** 10.3390/ijerph18136703

**Published:** 2021-06-22

**Authors:** Diego Fernández-Lázaro, David Gallego-Gallego, Luis A. Corchete, Darío Fernández Zoppino, Jerónimo J. González-Bernal, Blanca García Gómez, Juan Mielgo-Ayuso

**Affiliations:** 1 Department of Cellular Biology, Histology and Pharmacology, Faculty of Health Sciences, University of Valladolid, Campus of Soria, 42003 Soria, Spain; david_gallego12@hotmail.com; 2 Neurobiology Research Group, Faculty of Medicine, University of Valladolid, 47005 Valladolid, Spain; 3 Network Center for Biomedical Research in Cancer (CIBERONC), 37007 Salamanca, Spain; lacorsan@usal.es (L.A.C.); dfx@ubu.es (D.F.Z.); jejavier@ubu.es (J.J.G.-B.); jfmielgo@ubu.es (J.M.-A.); 4 Department of Health Sciences, Faculty of Health Sciences, University of Burgos, 09001 Burgos, Spain; 5 Department of Business Organization and Marketing and Market Research, Faculty of Business and Labor Sciences, University of Valladolid, Campus of Soria, 42003 Soria, Spain; bgarcia@eade.uva.es

**Keywords:** sports performance, ergogenic aids, respiratory muscles, inspiratory muscle training, pulmonary function, PowerBreath^®^

## Abstract

This systematic review and meta-analysis aim to provide scientific evidence regarding the effects of training on respiratory muscle training’s impact with the PowerBreath^®^. A systematic analysis based on the *PRISMA* guides and a conducted research structured around the bases of Web of Science, Scopus, Medline/PubMed, SciELO y Cochrane Library Plus. Six articles published before January 2021 were included. The documentation and quantification of heterogeneity in every meta-analysis were directed through Cochran’s Q test and the statistic I^2^; additionally, a biased publication analysis was made using funnel plots, whose asymmetry was quantified Egger’s regression. The methodological quality was assessed through McMaster’s. PowerBreath^®^ administering a ≥ 15% resistive load of the maximum inspiratory pressure (PIM) achieves significant improvements (54%) in said pressure within 4 weeks of commencing the inspiratory muscle training. The maximal volume of oxygen (VO_2_max) considerable enhancements was achieved from the 6 weeks associated with the maximum inspiratory pressure ≥ 21.5% post inspiratory muscle training onwards. Conversely, a significant blood lactate concentration decrement occurred from the 4th week of inspiratory muscle training, after a maximum inspiratory pressure ≥ 6.8% increment. PowerBreath^®^ is a useful device to stimulate sport performance and increase pulmonary function.

## 1. Introduction

The main function of the respiratory system is to maintain alveolar ventilation (oxygen-(O_2_) intake) in proportion to the metabolic needs of the organism, which increase during physical activity (PA) [1]. Also, carbon dioxide (CO_2_) exhalation is the main driver of ventilation to prevent arterial blood carbon dioxide pressure (PaCO_2_) from increasing and arterial blood oxygen pressure (PaO_2_) from decreasing [2]. During an intense and prolonged PA, the muscular endurance of the respiratory tracts decreases as a response to an increase in the respiratory muscle work and dyspnoea [3]. This fact induces fatigue in the respiratory muscles (RM) and reduces the respiratory function, resulting in a lessening of respiratory endurance [4]. This reduced respiratory activity could be connected to the activation of the metabolic reflex mechanism of the respiratory muscles (RMRM) “*metaboreflex*”. The RMRM is initiated by the fatigue of the respiratory muscles, which, through the afferent pathways III and IV, reaches the supraspinal level, causing a sympathetic vasoconstrictor response in the locomotor peripheral musculature which intensifies the fatigue of the active muscles and, in addition, increases the perception of effort, contributing to an endurance limitation linked to intense aerobic exercise [5]. Furthermore, the respiratory fatigue prevents the RM from reaching a suitable pleural pressure, this is an endurance limiting factor especially in disciplines which require aerobic resistance [6]. Other limiting factors of high-intensity physical endurance are pulmonary mechanics and pulmonary diffusion themselves [3].

Elite athletes of diverse modalities tend to combine ergogenic strategies in the hopes of improving their physiological responses and their competitive endurance; however, scientific evidence is occasionally limited [7]. One of the strategies employed is inspiratory muscle training (IMT), whose purpose is to enhance exercise tolerance [8]. IMT has been used to minimize and/or delay respiratory fatigue, the RMRM and the blood lactate concentration (LA) [9]. In this way, IMT could be considered a training method with a potential ergogenic effect to improve athletic performance [10]. Additionally, it has been suggested that other physiological mechanisms could explain the ergogenic effect of the IMT: diaphragm hypertrophy, an increase of the sanguine flow to locomotor muscles, a diminution in the subjective blood flow, reduction of fatigue, decreased dyspnoea, an increment in the efficiency and respiratory endurance, an alteration in the composition of muscular fibres to type I and augmentation of fibres type II in intercostal muscles, optimization of neuro-motor control in respiratory muscles maintaining the production of pressure with a minor motor impulse and a higher economization of the respiratory muscles [11]. Moreover, IMT is employed as a treatment for patients with respiratory condition—such as asthma, dyspnoea, and chronic obstructive pulmonary disease—with a better standard of living of the patients as a result [12,13].

IMT devices, which perform sectorized training of the respiratory muscles, can be divided into three categories: of resistive charge, of voluntary isocapnic hyperpnea, and threshold devices [14]. PowerBreathe^®^ (PwB) [PowerBreathe International Ltd. Southam, Warwickshire; England UK] is a threshold device that allows air flow during an inspiration only after reaching a certain inspiratory pressure, which is adjustable through the tension of a spring in accordance with the maximal inspiratory pressure (MIP) of a patient. Once this pressure is surpassed and the valve is opened, the lineal resistance to the flow increment must be inappreciable [15]. The PwB device enables higher charges between 186 and 274 centimetres of water (cmH_2_O) pressure to be generated by the lungs due to the force of the inspiratory muscles [3].

The inclusion of new elements in PA routines and/or training routines has recently been carried out by both professional and recreational athletes, with the aim of establishing adjustments that would turn into a differential element in their performance [3]. Nevertheless, the results are contradictory, because while IMT has proved to be effective in team sports [4,9,16], cycling [11,17] and runners [18,19], in other studies its efficiency has not been proved [7,20]. Discrepancies could be a result of the methodology (intensity and/or duration of the exercises), the design of the studies and the athletic expertise of the individual employing IMT. Likewise, it is important to consider the kind of improvements attainable in relation to physical endurance. Considering these circumstances, we decided to execute a systematic revision of these practices to critically evaluate the effects of IMT on respiratory parameters and athletic performance. A PwB device was employed on people who practice diverse types of physical activities. This study describes the magnitude of the inspiratory resistance, the frequency, and the duration of the IMT to establish an optimal programme which will allow improvements in respiratory and athletic endurance.

## 2. Methods

### 2.1. Search Strategy

This systematic review with meta-analysis focuses on analysing the impact of IMT in the athletic endurance of physically active subjects through a PwB device. The study was conducted following the *Preferred Reporting Items for Systematic Review and Meta-Analyses* (PRISMA) guidelines. To select the studies, the PICOS question model was used as follows: P (population) “physical active practitioners”, I (intervention): “respiratory muscle training through the PwB device”, C (comparators): “some conditions with/without PwB”, O (outcome): “use protocol, employed methodology, respiratory parameters and sports performance” and S (studies design): “random design with/without placebo” [21].

A bibliographical structured research was carried out through the online search of original articles of three literary data bases (Web of Science, Scopus, Medline/PubMed, SciELO and Cochrane Library Plus). The search included original articles written in various languages and published before 31 January 2021. Apart from small variations in the mechanisms of the said databases, the same chain of research was used in every record. Search terms were a mix of medical subject headings (MeSH) and key words related to Powerbreath, inspiratory muscle training, exercise, and athletic performance. They were the following: (“Powerbreath”) AND (“muscle” OR “inspiratory muscle training” OR “inspiratory muscle strength”) AND (“performance” OR “athletic performance”) AND (“exercise” OR “physical activity” OR “aerobic capacity” OR “resistance”). Throughout this research, relevant articles were acquired by applying the snowball strategy. Every title and summary of the research were intersected to identify published articles and potential studies missing. The titles and summaries were examined afterwards for a later revision of the full text. The authors completed their investigation of the studies published independently and the discrepancies in relation to the physical parameters were resolved through a discussion. This process was conducted by two investigators (D.F.-L. and J.M.-A.), who discussed discrepancies, and any disagreements were resolved by third-party evaluation (D.G.G.).

### 2.2. Selection of Articles: Inclusion and Exclusion Criteria

To select the studies employed, the following inclusion criteria were applied: (1) Documents should represent a well-designed experiment which includes respiratory musculature in a physical activity program via a PwB device in physically active subjects; (2) An identical situation in the subjects not employing a PwB; (3) Documents with no deadline restriction; (4) publications whose study subjects were physically active humans; (5) Specific information related to PwB and physical activity; (6) Languages were limited to English, German, French, Italian, Portuguese and Spanish.

Concerning the exclusion criteria, the following studies were not regarded: (1) Documents not related to inspiratory muscle training with PwB; (2) Physically inactive individuals or subjects with comorbidities which would avert respiratory training or the practice of physical activity; (3) Duplicated documents; (4) Experiments which were not performed in humans; (5) Studies that consisted of systematic or narrative revisions, editorials, letters to the editor or commentaries.

### 2.3. Data Extraction

With the inclusion/exclusion criteria having been applied to every study, the data of the research source, including authors and date of publication, the participants’ characteristics, the research design, the PwB device model employed, the inspiratory muscle training routine, the parameters analysed, and the results and conclusions of the experiments, were extracted independently by the authors using a spreadsheet (Microsoft Inc, Seattle, WA, USA). Subsequently, the discrepancies were resolved by having two authors (D.F.-L. and J.M.-A.) discuss among themselves until a consensus was reached.

### 2.4. Data Analysis

Firstly, we proceeded to identify and quantify the heterogeneity of our data via Cochran’s Q test and the I^2^ statistic. A *p*-value < 0.05 in the Q test was considered as a proof of the rejection of the null hypothesis regarding the homogeneity of the experiments. Additionally, I^2^ values over 25%, 50% and 75% were selected to represent low, moderated, and high heterogeneity, respectively. Based on the results of these heterogeneity tests, we performed a fixed-effect meta-analysis when the absence of heterogeneity was proved. Otherwise, a random effects meta-analysis model was employed. The variance among the studies in the random effects meta-analysis, also known as tau squared (τ2), was calculated using DerSimonian-Laird’s method [22]. The effect size (ES) was estimated as the logarithmic transformed Ratio of Means (ROM) of the PwB and placebo groups. A z-test was implemented to determine the significance of the ES. Finally, a publication bias analysis was performed using funnel plots; these graphics’ asymmetry was quantified employing the Egger’s regression [23]. This bias analysis was carried out via the “Trim and fill” method. All the meta-analysis workflow was performed using the metaphor package (version 2.1-0) in R (The R Foundation for Statistical Computing, Vienna, Austria).

### 2.5. Quality Assessment

The methodological quality evaluation of the selected articles was assessed using the McMaster’s Critical Review Form [24]. The aim of this evaluation was to exclude studies with poor methodology, with a score less than or equal to 10 points. The methodological quality of the selected studies was assessed by the same two authors (D.F.-L. and J.M._A.), and any disagreements were resolved by third-party evaluation (D.G.G.).

## 3. Results

### 3.1. Selection of Studies

**Figure 1:**
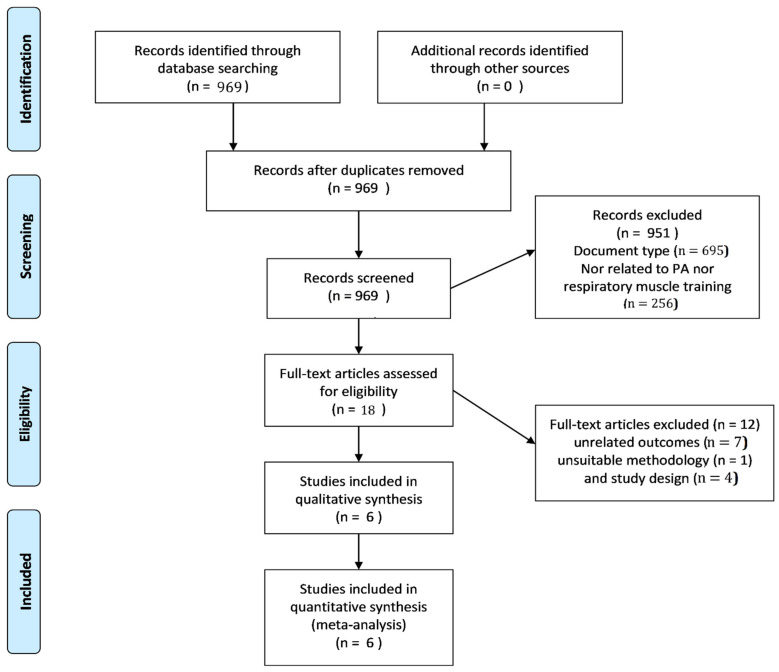
Preferred Reporting Items for Systematic Reviews and Meta-Analyses (PRISMA) flow diagram study selection process for the systematic review.

We identified an initial total of 969 records of articles published after 2007. Among those, 695 were rejected for the lack of intervention and 256 because of not being related to the research topic. We also excluded 12 articles after full-text review. Reasons for exclusions after full-text review were unrelated outcomes (*n* = 7), unsuitable methodology (*n* = 1) and study design (*n* = 4). The remaining six studies (n = 6) [4,9,11,16,19,25] met our inclusion criteria and were included in the present systematic review (Figure 1).

### 3.2. Descriptive Information of the Selected Articles Included in the Systematic Review

The characteristics of the studies included in the systematic review appear in Table 1.

### 3.3. Results of the Quality Assessment

Then we conducted a quality assessment of the articles. The scores of the selected articles ranged from 12 to 14 points. Five studies were *“very good”* and one was *“good”*. No studies were excluded because of poor quality. Details about the results of the quality assessment are shown in Table 2.

### 3.4. Performance Measures

Table 3 summarizes the studies included in the present review. Table 3 displays information about the authors, publication year, study design, population, the type of PwB device employed, the training protocol, the analysed parameters, the results, and the final conclusions.

### 3.5. Meta-Analysis

#### 3.5.1. Effect on Maximal Inspiratory Pressure (MIP)

**Figure 2:**
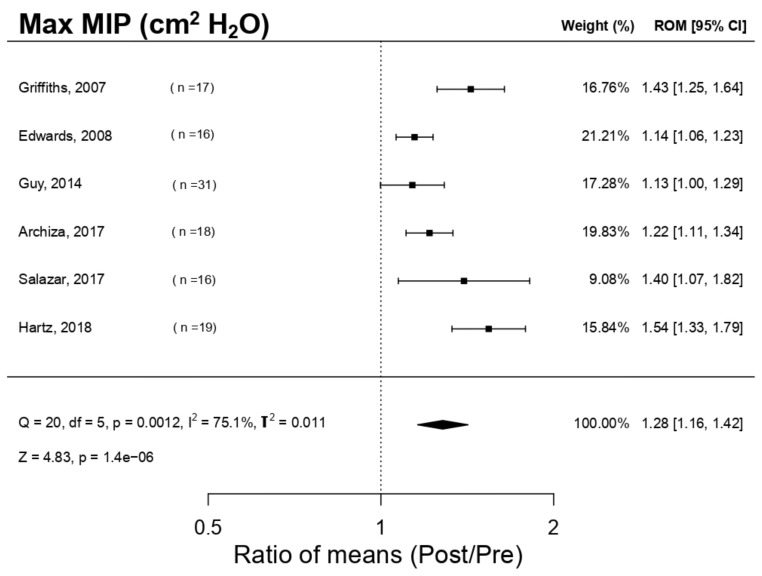
Forest plot of the meta-analysis of the effects of inspiratory muscle training with PowerBreath^®^ on Peak Inspiratory Pressure. MIP = Peak Inspiratory Pressure; CI = Confidence Interval; ROM = Ratio of Means; Pre = Pre-inspiratory muscle training; Post = Post-inspiratory muscle training; Q = Cochran’s Q statistic; df: degrees of freedom; p = *p*-value (the first one refers to Cochran’s Q, the second one to Z); I^2^: I-square statistic; T^2^: Tau-squared; Z: Z-value.

**Figure 3:**
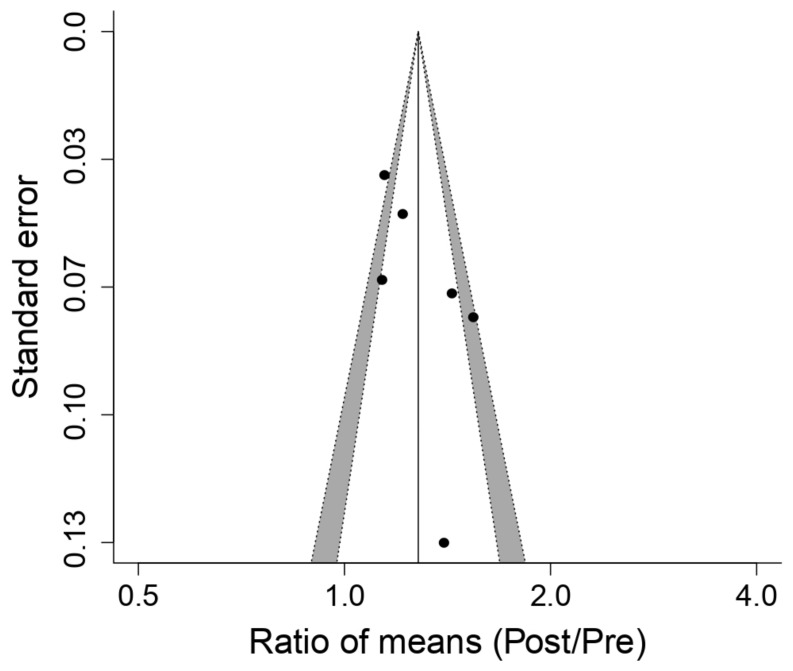
Funnel of the meta-analysis of the effects of inspiratory muscle training with PowerBreath^®^ on Peak Inspiratory Pressure. Pre: Pre-inspiratory muscle training; Post: Post-inspiratory muscle training.

Figure 2 shows the effect of respiratory muscle training with a PwB device over maximal inspiratory pressure (MIP) and indicates that said device produces an enhancement effect that is statistically relevant (*p* = 1.4 × 10^−6^) over MIP: ROM 1.28; 95% IC, 1.16 a 1.42; Q = 20, df = 5, *p* = 0.0012, I^2^ = 75.1%, T^2^ = 0.011; Z = 4.83, *p* = 1.4 × 10^−6^. The investigations [4,9,11,16,19,25] examined in the meta-analysis presented substantial increments in MIP. The following publication bias analysis (Figure 3), not having detected a significant publication bias statistically (Egger *p*-value = 0.111), showed a relatively symmetrical funnel plot with respect to MIP.

#### 3.5.2. Effect on Maximal Oxygen Volume (VO_2_max)

**Figure 4:**
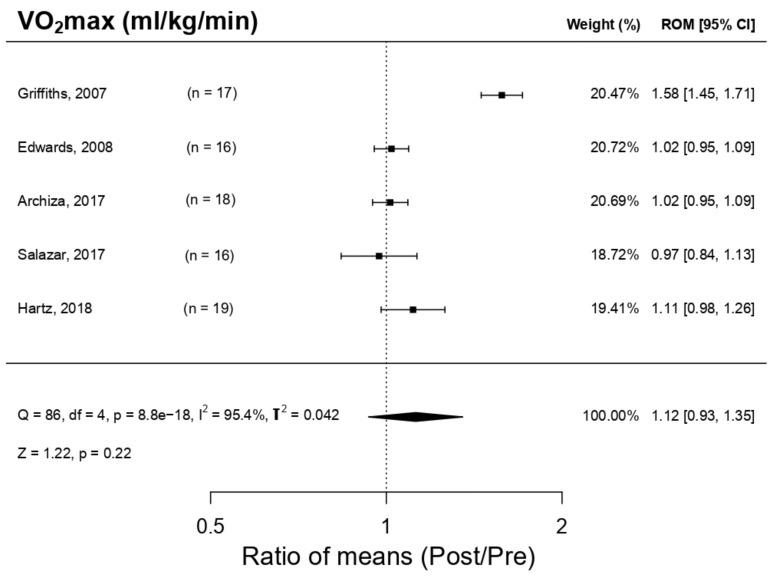
Forest plot of the meta-analysis of the effects of inspiratory muscle training with PowerBreath^®^ on Maximal oxygen volume. VO_2_max = Maximal oxygen volume; CI = Confidence Interval; ROM = Ratio of Means; Pre = Pre-inspiratory muscle training; Post = Post-inspiratory muscle training; Q = Cochran’s Q statistic; df: degrees of freedom; p = *p*-value (the first one refers to Cochran’s Q, the second one to Z); I^2^: I-square statistic; T^2^: Tau-squared; Z: Z-value.

**Figure 5:**
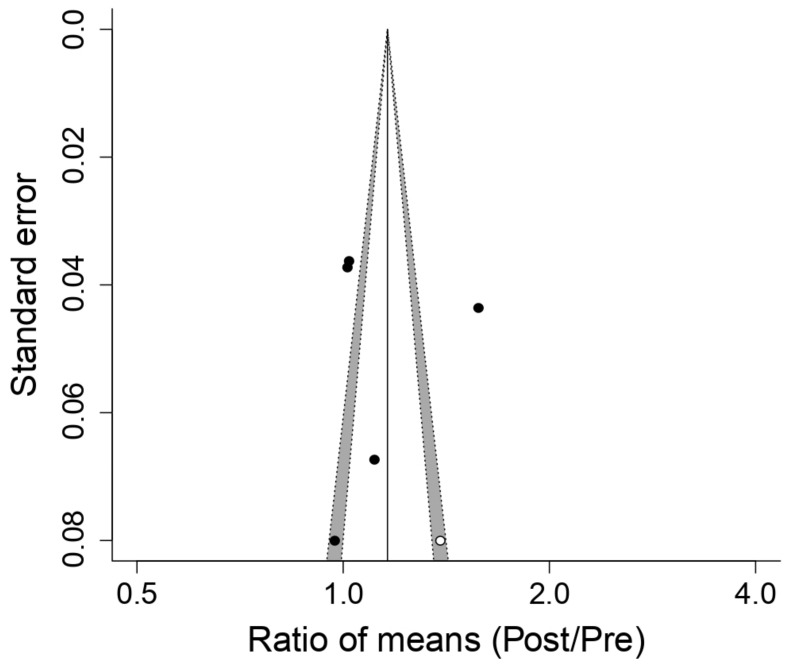
Funnel plot of the meta-analysis of the effects of inspiratory muscle training with PowerBreath^®^ on Maximal oxygen volume. Pre: Pre-inspiratory muscle training; Post: Post-inspiratory muscle training.

Figure 4 illustrates the training effect in respiratory muscles with a PwB device on maximal oxygen volume (VO_2_max) and indicates that the device can lead to significant enhancements in VO_2_max, despite the pre-established threshold of statistical significance in the *p*-value < 0.05→ ROM 1.12; 95% IC, 0.93 a 1.35; Q = 86, df = 4, *p* = 8.8 × 10^−^^18^, I^2^ = 95.4%, T^2^ = 0.042 Z = 1.22, *p* = 0.22 not being reached. Salazar et al. [11] were the only authors included in this review to observe a VO_2_max reduction; the remaining studies [4,9,19,25] observed an increment in VO_2_max; Griffiths et al. [25] presented a notable increment, with a 1.58 ROM (95% IC: 1.45–1.71), even though, in this analysis, no publication bias was detected on the basis of Egger’s regression (*p*-value = 0.672). By employing the trim and fill method, an imputation of a study at higher levels of ES took place with a standard error—this could point towards the lack of studies at this level (Figure 5).

#### 3.5.3. Effect on Blood Lactate Concentration (LA)

**Figure 6:**
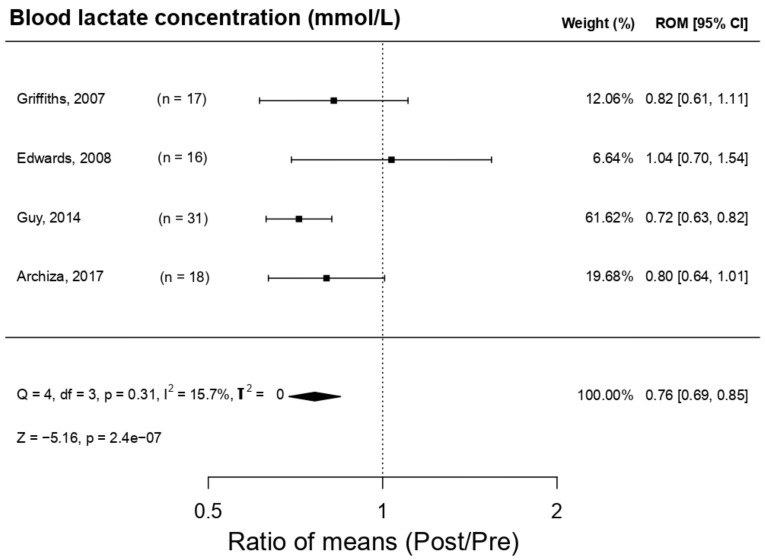
Forest plot of the meta-analysis of the effects of inspiratory muscle training with PowerBreath^®^ on blood lactate concentration. CI = Confidence Interval; ROM = Ratio of Means; Pre = Pre-inspiratory muscle training; Post = Post-inspiratory muscle training; Q = Cochran’s Q statistic; df: degrees of freedom; p = *p*-value (the first one refers to Cochran’s Q, the second one to Z); I^2^: I-square statistic; T^2^: Tau-squared; Z: Z-value.

**Figure 7:**
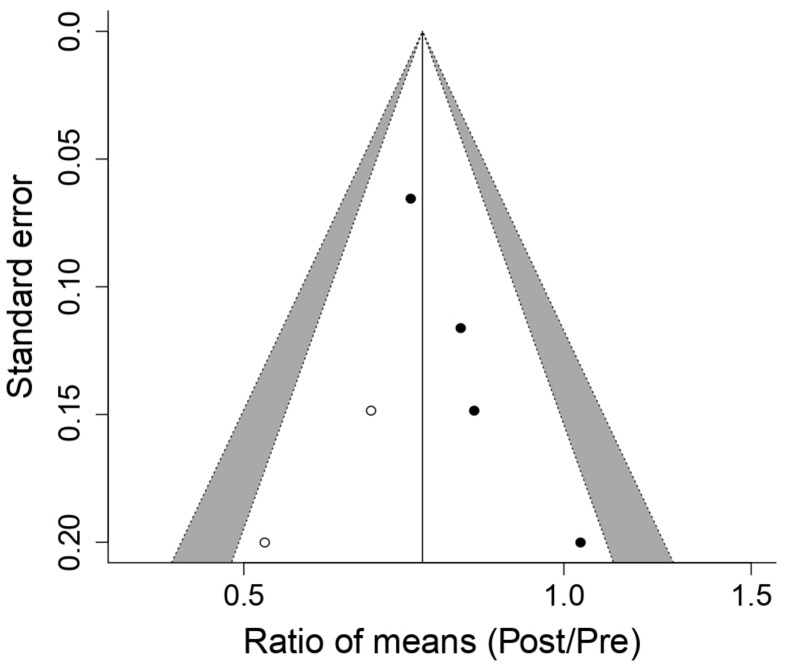
Funnel plot of the meta-analysis of the effects of inspiratory muscle training with PowerBreath^®^ on blood lactate concentration. Pre: Pre-inspiratory muscle training; Post: Post-inspiratory muscle training.

Figure 6 displays the effect of respiratory muscle training with a PwB device on lactate concentration (LA). The outcome of the meta-analysis indicates that the PwB device produces a statistically significant decrement (*p*-value = 2.4 × 10^−7^) in lactate concentration post-training compared to pre-training: → ROM 0.76IC 0.69 a 0.85; Q = 4, df = 3, *p* = 0.31, I2 = 15.7%, T2 = 0, Z = −5.16, *p* = 2.4 × 10^−^^07^. In three [9,16,25] out of the four studies [9,16,19,25], a diminution in LA levels were observed post-training; only Edwards et al. [19] broke this tendency. Nevertheless, this study presented a high internal heterogeneity, as demonstrated by its wide confidence interval of 95% (0.70–1.54); hence, its weighing in the meta-analysis was lower. Lastly, a publication bias study was carried out through funnel plots (Figure 7). Via the trim and fill method, a possible publication bias was detected because of the imputation of two cases where ROM levels were lower than the ES; this indicates the possibility of the omission of studies in which the reduction of LA concentration would be more acute. Despite this result, the study of the graph’s asymmetry based on Egger’s regression indicated that this omission was not statistically significant (*p*-value = 0.067).

## 4. Discussion

The main objective of this investigation was to analyse the scientific evidence to assess the effects of IMT through the usage of a PwB device in individuals who practice PA of various sorts. In this way, it was concluded that the usage of a PwB device applying a resistive charge ≥ 15% of the MIP makes it possible to improve overall physical endurance in the diverse athletic fields in which it was implemented. The highest percentual increments of the MIP (54%) were achieved after 12 weeks, although significant improvements of said MIP were observed after 4 weeks. Regarding the performance parameters assessed, substantial enhancements of VO_2_max were attained after 6 weeks with respect to the increment of MIP ≥ 21.5% post-IMT. On the other hand, a considerable decrement of LA was achieved from 4 weeks of IMT onwards, with an increment of MIP ≥ 6.8%. With the aim of providing a more evident analysis, the variables included in this revision were organised as follows.

### 4.1. Athletic Methods and PowerBreath^®^ (PwB) Models

The PwB Plus Heavy [4], PwB K1 [16], PwBK3 [11] and PwBK5 [9] devices were the various models of PwB employed in this investigation. Although different PwB models were used, all of them achieved the indicated IMT load, thus avoiding a possible risk of bias in the analysis. The PwBK5 version was the only one that facilitated real-time monitorization and provided software through which easy-to-analyse records could be downloaded [3]. IMT using a PwB device was applied in athletic routines of various fields, such as handball [4], rowing [25], cycling [11], athletics [19] and football [9,16]. In each one of these activities, divergent physical and metabolic components play a role [3]. This could render PwB devices a relatively new multimodal training method that encompasses many physiological patterns within a single respiratory training session; the latter could establish beneficial modifications in respiratory parameters and aerobic and/or anaerobic performances. The usage of a PwB device was present in all the studies analysed in this paper; however, the series, versions and models employed were dissimilar; in some cases, these data were not even specified [4,9,11,16,19,25].

### 4.2. PowerBreath^® ^(PwB) Appliance Method

It is important to consider and precisely determine the fundamental principles of the selection of the charge—including the level of the inspiratory resistance applied, its frequency and the duration of the training units, with its placement within the training cycle of the athletic field—as the effects of IMT on RM are contingent on these principles [26]. Recent studies surrounding IMT and its repercussion on health [12,13,27,28], as well as its athletic performance [7,20,26] and the articles included in this study [4,9,11,16,19,25], follow the recommended guidance of PwB [3], which recommends the execution of 30 maximal inspirations twice a day to specifically strengthen inspiratory muscles and obtain the benefits derived from said fortification as health and/or physical endurance improvements.

The 30 inspirations procedure can be justified since IMT is strength training and, as such, it should not be different in terms of its methodology and effects in relation to generic strength training programmes for skeletal musculature [29]. Additionally, there is a converse relation between the maximum strength percentage and the number of repetitions that can be achieved. Therefore, the IMT based on 30 maximal inspirations could be a simple and general way of establishing the intensity of the strength performance corresponding to, approximately, 50% of the maximal strength, which, in this case, would be like the MIP. Once the patient is able to complete the recommended 30 repetitions comfortably, the inspiration resistance—the mechanical resistance in the PwB—is augmented, making it possible to adapt the resistance and the IMT to the progressively acquired improvements [3]. Alternatively, the usage of the PwB device can be used to complement the training routine in specific athletic fields for a duration of 4–12 weeks of IMT with a resistive charge between 15–70% MIP [4,9,11,16,19,25]. The producer’s guidelines establish that the resistive charge of the IMT should be >30% of the MIP and, according to what was described by HajGhanbari et al. [6], a charge <15% of the MIP is ineffective. Among the investigations analysed, only Edwards et al. [19] used a fixed charge of 15% of MIP; the rest of the studies employ constant values of either 50% [9,11,25] or 55% [16] or they apply a gradual methodology, between 50–70% of the MIP, according to [4].

### 4.3. Ergo-Respiratory Parameters Evaluation

The resistance of the RM can be trained and, after implementing an appropriate program, can influence the energetic metabolism of the RM becoming more efficient and causing a lesser demand of O_2_ in respects to the skeletal musculature. In addition, the strength of the RM increases. At the same time, it is difficult to determine which factors diminish fatigue and/or improve the final endurance, and to what extent [30]. This is due to the fact that, because of the location and function of the RM, it is complicated to evaluate and quantify their fatigue; despite this, there are respiratory functional parameters which are modified during the IMT [6,15]. In this way, the articles included in this revision, in order to carry out an optimal control of the IMT, evaluated various respiratory parameters: MIP [4,9,11,16,19,25], maximal expiratory pressure (MEP) [6,25], respiratory effort ratio (RER) [25], respiratory efficiency (RE) [11], ventilation/minute [9], forced vital capacity [16], and forced expiratory volume in a second (FEV_1_) [9,11,16,19].

### 4.4. Maximal Respiratory Pressure

Concerning pulmonary ventilation, it has been proved that, after intense aerobic and/or long-duration physical exercises, significant decrements on the MIP and MEP occur [31] as a result of the strength and endurance reduction of the RM. These respiratory factors limit performance of the athletes [6], and thus IMT would be beneficial.

The MIP primarily evaluates the diaphragmatic force [32]. The results of the meta-analysis showed that IMT with a PwB device produces a significant improvement on MIP, which could lead to enhancements in the oxidative capacity of the diaphragm [33] and an increment of strength that infers greater resistance to fatigue [6]. Some authors [8,17] suggest that the improvement on the endurance is determined by changes >25% of MIP post-IMT. However, this was only asserted in half of the studies analysed [4,11,25], where the investigators obtained improvements of the MIP between 26 and 54%. The three remaining studies [9,16,19], with a range of MIP improvements between 6.8 and 21.5%, obtained athletic endurance enhancements as well. This would imply that the magnitude of the MIP modification is not the sole means for the obtention of performance improvements; the intensity level of the IMT’s inspiratory resistance could be another method for attaining improvements. Every study analysed employed levels of resistive charge > 15% MIP, as described by HajGhanbari et al. [6], who used said levels of intensity as the resistive charge. It should be underlined that the largest gains of MIP (54%) took place in programs of a longer duration (12 weeks) [4]. In the remaining studies [9,11,16,19,25], where the length of the experiment oscillated between 4 and 6 weeks, the duration was not differential to the modifications of MIP post IMT.

The MEP assesses the strength of the intercostal and abdominal respiratory muscles [32]. Hartz et al. [4] observed non-relevant increments (23%) of the MEP connected to physical aerobic endurance enhancements and significant increases in the maximum volume oxygen (VO_2_ max) of handball players after 12 weeks of IMT. Likewise, Griffiths et al. [25] detected improvements in VO_2_max, as well as anaerobic capacity, after enhancements of the MEP (31%) after 4 training weeks with professional rowers. Consequently, IMT could lead to a higher muscle strength in intercostal and/or abdominal muscles, generating a sustained contraction during the exercise, thus allowing sufficient ventilation and increasing the efficiency of the RM [34]. This grants a greater resistance to fatigue throughout the practice of a PA by reducing the O_2_ supply to the intercostal and/or abdominal muscles during exercise related to skeletal muscles [18]. Accordingly, the increments, even when not significant—23–31% PEM—seem to show effectiveness when obtaining aerobic endurance increases. When superior to 31%, said increments could lead to an improvement of aerobic and anaerobic endurance.

Subsequently, the RM can be trained through resistance changes as well as monitored by functional respiratory parameters. The observation of incremental modifications in these parameters, MIP and MEP, can facilitate the overcoming of the limiting factors on exercise, such as the energetic restraints of RM with respect to the skeletal musculature involved in PA; in addition to the fatigue of the RM itself, here IMT can incite improvements [33].

### 4.5. Evaluation of Physical Parameters 

The VO_2_max measures the amount of O_2_ the body inhales and uses while performing PA. With VO_2_max intensities between 70 and 80%, the increment of LA becomes considerable, to produce power and energy are associated with the ATP resynthesis procedures of the anaerobic pathways, allowing high levels of VO_2_max to be achieved [35]. These markers, VO_2_max and LA, permit the monitoring of aerobic and anaerobic training capacities, respectively [36].

The result of the VO_2_max meta-analysis showed that IMT with PwB produces substantial improvements over this parameter, although these are not statistically relevant. This could be explained by the possible presence of a publication bias in said meta-analysis, which, as stated in the results section, could mean there have been no studies published with a result favourable to the increment of VO_2_max post-training, either because of the lack of research regarding this aspect or because of the omission of results. Nevertheless, it is notable that in the present revision of the four studies analysed [4,9,19,25] a VO_2_max increase was presented, particularly in Griffiths et al.’s case [25], where a considerable increment in this parameter was observed (1. 58 ROM). Thus, while waiting for a larger number of studies including an analysis of VO_2_max, we could consider the decreased modulation in the functionality of the RM during PA, as it increases the VO_2_max, potentially through triggering a delay in the RMRM and augmenting respiratory endurance.

It has been estimated that, during intense exercise, the RM could use 16% of the cardiac output, reducing the O_2_ availability for skeletal muscles, which oversee mobility [37]. This would occur due to the vasoconstrictor sympathetic response, which reduces the sanguine flow of the skeletal muscles, and therefore their energy production and consumption. As a result, the fatigue of the skeletal muscles increases, and the flow is redistributed to preserve the respiratory function without compromising the energetic demand of the RM, in favour of the respiration [5].

This situation positions the respiratory system as limiting for the VO_2_max and the performance of PA. Through an adequate IMT, the RM could potentially improve their fatigue tolerance, improve their respiratory efficiency, and delay the RMRM [38]. Otherwise, IMT could have a positive influence on physical performance by achieving significant VO_2_max improvements through resistance training intensities between 50 and 70% MIP and a duration of 6–12 weeks; these could lead to improvements of MIP >20% post IMT [6,9].

Similarly, in the training of RM using a PwB device, a statistically significant decrement in LA post-training was observed. The reduction of LA could be more acute, because based on the trim and fill method, a possible publication bias was observed in the funnel plot when imputing two studies whose ROM levels were lower than the ES. Therefore, under these circumstances, it could be argued that the IMT with PwB provides an advantage over the performance of athletes greater than that observed in the present paper, the inverse correlations between the increment of the respiratory function and the sanguine flow to the muscles, provoke the respiratory function during a maximal exercise to compromise the perfusion and VO_2_max of the skeletal muscles [37,39]. At high PA intensities, the production of LA increases rapidly because the pace at which the muscular glycogen is used is high and exceeds the mitochondrial rhythm to accept the private for its oxidation in the Krebs cycle [3]. Nevertheless, after the improvement in the aerobic capacity showcased post-IMT, the greater quantity of O_2_ brought to the muscles per time unit delays the appearance of the lactate threshold. Hence, the significant reduction of LA is associated with the improvements of VO_2_max [9,19,25]. The effectiveness of IMT was, then, presented through the significant LA reduction, with resistance training intensities between 15 and 50% MIP and a duration of 4–6 weeks, which were related to adaptations of 6.8% MIP post IMT.

Overall, one could articulate that, after training the RM, it is possible to observe improvements in the aerobic metabolism, alongside enhancements of the VO_2_max and an anaerobic decline of LA. These results could reveal that IMT produces an enhancement in fatigue tolerance and a greater respiratory efficiency, as stated by Mc Connell et al. [40]. Overall, these conclusions make it possible to position PwB as a device that assists in the completion of the customary training system of an athlete.

## 5. Application of PowerBreath^®^ (PwB) as a Non-Nutritional Supplement in Sports

PAs are limited because of the respiratory muscular function or ventilation after the practice of elongated sub-maximal exercises and short maximal exercises which produce fatigue in the RMs [41]. The results prove that RMs can be trained through specific training methods, such as IMTs using a PwB device, and are able to overcome these respiratory limitations. In this paper, even though divergent IMT protocols with PwB devices were described, and various responses to respiratory parameters—MIP, MEP—and exercise-control parameters—VO_2_max, LA—were reported, optimal results in final athletic endurance were produced; the results were specific to the physical activities [4,9,11,16,19,25]. These sports—handball [6], rowing [25], cycling [11], athletics [19] and football [9,16]—require maintaining high levels of intensity for long periods of time and the increment of strength in the RM post IMT can therefore lead to a higher resistance to the characteristic fatigue of the RM; this could be efficient in terms of acquiring net physical endurance [20]. In addition, the increment in the strength of these RM would make it possible to satisfy the ventilatory demand, which is the only way of eliminating the excessive metabolic production of CO_2_ during a PA, and which, if not heeded, could cause the CO_2_ to accumulate in the blood and tissues, triggering metabolic acidosis and, consequently, the breakdown of skeletal and respiratory musculature [42]. It has been estimated that the increase in strength commences from a 15% MIP increment onwards [16]; however, in Edwards et al. [19], with a significant MIP enhancement in athletes of 6.8% post-IMT, the benefits in the 5000 m test improved significantly. This fact complicates the interpretation of the improvements, establishing a MIP improvement threshold. Nonetheless, employing a fixed resistive charge of IMT ≥ 15% of the MIP with a PwB induces positive adjustments in the MIP, which could be directly linked to the overall physical endurance enhancements in the diverse PA fields. Concerning the duration of the IMT, the biggest percentual improvements of the MIP (54%) were produced after a 12-week period [4], even though significant enhancements of the MIP were observed after 4 weeks [25]. The substantial improvements of the VO_2_max were attained from 6 weeks onwards, with increments of the MIP ≥ 21.5% post-IMT [8]. A considerable reduction of LA was detected after 4 weeks of IMT, with the increments of MIP being ≥ 6.8% [19].

## 6. Conclusions

The results presented in this paper would qualify the PwB device as an efficient, easily manageable, and applicable to conventional training routines in various sporting fields; this fact would endorse the usage of IMT to stimulate endurance enhancements in the performance of physical activities.

However, to consolidate the conclusions of the current paper, it is recommended to carry out a wider range of studies analysing the influence of IMT on endurance control parameters, most specifically those which estimate VO_2_max and LA, as a potential publication bias with respect to these two areas was confirmed. Lastly, to establish a suitable IMT method based on the components of each PA, which would positively affect respiratory functions and would redound the physical activity directly, is advised. Sports medicine, for its comprehension in mechanisms that would solidify IMT results, will oversee the design of a unique programme tailored to the PA and its ventilatory demands for incorporation into an individual IMT regime. 

## Figures and Tables

**Table 1:** Descriptive synthesis of the studies included in the systematic review.

Subject’s level	Professional	3 studies [4,9,25]
	Amateur	3 studies [11,16,19]
Subject’s age	Senior (20–30)	5 studies [4,9,11,16,25]
	N/A	1 study [19]
Training method	2 daily sessions, 5 days/week (30 inspirations 50% MIP)	3 studies [4,9,11]
	4 weeks IMT + 6 combined weeks (IMT + EMT)	1 study [25]
	2 running weekly sessions + IMT (30 inspirations)	1 study [19]
	2 days/weeks regular football training + IMT (2 times/day 30 inspirations at the subject’s own pace)	1 study [16]
	4 weeks	1 study [19]
	6 weeks	3 studies [9,11,16]
	10 weeks	1 study [25]
	12 weeks	1 study [4]

MIP: maximal inspiratory pressure; IMT: inspiratory muscle training; EMT: expiratory muscle training; IM: inspiratory musculature.

**Table 2:** Results of the quality assessment.

Reference	ITEMS					T_E_					%						MC		
	1	2	3	4	5	6	7	8	9	10	11	12	13	14	15	16			
Hartz el al. 2018 [4]	1	1	1	1	1	1	1	1	1	1	0	0	1	1	1	1	14	87.5	VG
Griffiths et al. 2007 [25]	1	1	1	1	0	1	1	1	1	1	1	0	0	1	1	0	12	75.0	G
Salazar-Martínez et al. 2017 [11]	1	1	1	1	0	1	1	1	1	1	1	1	0	1	1	0	13	81.3	VG
Edwars et al. 2015 [19]	1	1	1	1	0	1	1	1	1	1	1	0	1	1	1	1	14	87.5	VG
Archiza et al. 2017 [9]	1	1	1	1	0	1	1	1	1	1	1	1	0	1	1	0	13	81.3	VG
Guy et al. 2014 [16]	1	1	1	1	1	1	1	1	1	1	0	0	1	1	1	1	14	87.5	VG
T	7	7	7	7	2	7	7	7	7	7	5	1	3	7	7	3			

(T) Total items achieved. (T_E_) Total items/study (1) Accomplished criteria; (0) Unaccomplished criteria MC: Methodological quality [Low ≤8 points; Acceptable (A) 9–10 points; Good (G) 11–12 points; Very Good (VG) 13–14 points; Excellent (E) ≥15 points].

**Table 3:** Summary of the results of the studies included in the systematic review.

Characteristics of the Studies Included in the Systematic Review							
Authors/Year	Population	Study Design	PowerBreaht^®^	Respiratory Muscle Training	Analysed Parameters	Results	Conclusion
Hartz et al. 2018 [4]	19 ♂ (20 ± 3 yo) professional handball players. Group PwB: *n* = 10, 19 ± 4 yo Placebo group: *n* = 9, 22 ± 1 yo	Random, with Placebo	PwB Plus Heavy Resistance Sports Model	2 h/session 5 sessions/weeks For 12 weeks RES increment 50–70% MIP	MIP MEP MVV PP VO_2_ max	↑MIP * ↑MEP ↑MVV ↑PP ↑VO_2_max *	IMT produces a relevant increment in the strength and resistance and, therefore, endurance
Griffiths et al. 2007 [25]	17 ♂ professional rowers. Group A: (*n* = 10; 24.9 ± 5.6 yo). Gruop B: *n* = 7; age, 28.7 ± 9.1 yo)	Random, with Placebo	Group A: IMT PwB Group B: EMT Powerlung (IMT inactivated)	4 weeks; 30 respirations * 2/day Group A: IMT Group B: EMT RES 50% MIP	MIP MEP HR VO_2_ max LAB RRE	↑MIP * ↑MEP HR X ↑VO_2_ max * ↓LAB * ↓RRE	IMT (using PwB) improved the performance of rowers, but EMT (using Powerlung) does not.
Salazar-Martínez et al. 2017 [11]	16 amateurs’ cyclists (23.05 ± 4.7 yo). 9 ♂ (23.44 ± 2.7) and 7 ♀ (25.37 ± 3.24)	Random, controlled, without Placebo	PwB K3	6 weeks; 30 respirations * 5 days/week; 2 sessions/day. RES 50% MIP	MIP ER En VO_2_ max FEV1 OUES	↑MIP * ↑ER ↑En * ↓VO_2_ max * ↓FEV_1_ * ↑OUES *	IMT has a positive effect in the performance of cyclists
Edwards et al. 2015 [19]	16 ♂ endurance amateur athletes	Random, with Placebo	PwB	4 weeks; in each week: IMT: 30 respirations * 1 session/day RES 15% MIP + (CV1: 5 × 1000 m; CV2: 3 × 1600 m, SP1: 20′ running)	MIP VO_2_ max HR LAB RPE Test duration 5000 m FEV1	↑MIP * VO_2_ max X HR X ↓ LAB ↓ RPE * ↑Test duration 5000 m * ↑FEV_1_	IMT combined with cardiovascular training increases the MIP, improves significantly capability/endurance in 5000 m test; it does not affect VO_2_max
Archiza et al. 2017 [9]	18 ♀ professional footballers. Group PwB: (*n* = 10; 22.0 ± 3.9 yo). Simulation Group: (*n* = 8; 20.1 ± 2.0 yo)	Double random blind controlled by a simulation (placebo).	PwB K5	6 weeks; 5 days/week; 30 respirations * 2 sessions/day RES50% MIP	Antropometrics, pulmonal function, respiratory musculature strength, incremental maximal exercise test + “Tlim” test + repeated sprints	↑MIP * ↓VE ↓(44)B * ↑RSA ↑VO_2_max * ↓FEV_1_	IMP increases the strength of inspiratory muscles, exercise tolerance and recovering process post repeated sprints in footballers
Guy et al. 2014 [16]	31 ♂ amateur footballers. Group PwB: *n* = 12; 28.4 ± 8.2 yo). Group Placebo: *n* = 9; 23.9 ± 6.7 yo). Control Group: *n* = 10; 21.3 ± 4.9 yo)	Random, with Placebo and control group	PwB K1	6 weeks 2 sessions/weeks of normal training + 2 * 30 inspirations IMT/session RES 55%	MIP FVC FEV1 MSFT LAB SSFT	↑MIP * FVC X ↓FEV_1_ ↑MSFT * ↓LAB * SSFT X	IMT increases the endurance of the footballers’ preseason

♂: Males; ♀: Females; *: Statistically significant; ↑: Increment; ↓: Decrement; X: No statistically relevant variations; PwB: PowerBreath^®^; yo: years old; MIP: Maximal Inspiratory Pressure; MEP: Maximal Expiratory Pressure; MVV: Maximal Ventilatory Pressure; PP: Physical Performance (physical aerobic performance); HR: Heart Rate; VO_2_max: Maximal oxygen volume; LAB: Max Lactate in Blood; RER: Respiratory Effort Ratio; IMT: Inspiratory Muscle Training; EMT: Expiratory Muscle Training; RE: Respiratory Efficiency; En: Endurance; CV1: Cardiovascular test 1 (5 × 1000 m); CV2: Cardiovascular test 2 (3 × 1600 m); SP1: Self-Paced (Running at one’s own pace for 20 min); Tlim: Time-to-exhaustion test (Exhaustion measurement test); VE: Ventilation/minute; m: meters.

## Data Availability

Not applicable.

## Abbreviations

O_2_	Oxygen
PA	Physical activity
RM	Respiratory muscles
RMRM	Metabolic reflex mechanism of the respiratory muscles “*metaboreflex*”
IMT	Inspiratory muscle training
LA	Blood lactate concentration
CO_2_	Carbon dioxide
MIP	Maximal inspiratory pressure
PwB	Power-Breathe
cmH_2_O	Centimetres of water
SL	Standard of living
PRISMA	Preferred Reporting Items for Systematic Review and Meta-Analyses
P	Population
I	Intervention
C	Comparators
O	Outcome
S	Studies design
MeSH	Titles of medical matter
*S* ^2^	Within-study variance
τ^2^	Tau squared
ES	Effect size
ROM	Ratio of Means
VO_2_max	Maximal oxygen volume
MEP	Maximal expiratory pressure
RER	Respiratory effort ratio
RE	Respiratory efficiency
FEV_1_	Forced expiratory volume in a second
